# The Frequency of Anatomical Variants of the Bile Ducts: A Review Based on a Single Classification as Support for Cholangiographic Examinations

**DOI:** 10.7759/cureus.58905

**Published:** 2024-04-24

**Authors:** Norman B Olmedo, José Sebastião Dos Santos, Jorge Elías Junior

**Affiliations:** 1 Department of Imaging and Radiology, College of Medical Sciences, Central University of Ecuador, Quito, ECU; 2 Department of Surgery and Anatomy, Hospital das Clinicas da Faculdade de Medicina de Ribeirão Preto, Universidade de São Paulo, Ribeirão Preto, BRA; 3 Department of Medical Imaging, Hematology, and Oncology, Ribeirão Medical School, University of São Paulo, Ribeirão Preto, BRA

**Keywords:** bile duct, iatrogenic bile duct injury, variant, anatomy, magnetic resonance imaging, bile ducts

## Abstract

Complications arising from hepatobiliary surgery can have adverse effects on both the quality of life and the survival of patients. Magnetic resonance cholangiography (MRC) techniques are highly effective at revealing anatomical variants of the bile ducts and thus play a vital role in minimizing the occurrence of complications.

The aims of this review are threefold: to ascertain the classifications utilized for categorizing anatomical variants of the bile ducts, to present the reported results on the prevalence of these anatomical variants, and to explore the diagnostic modalities employed to visualize these anatomical variants and associated complications during surgical procedures.

A review of the literature was carried out using the Cochrane Library database and the PubMed, Medical Literature Analysis and Retrieval System Online (MEDLINE), and Google Scholar platforms. We conducted a comprehensive review of relevant studies to categorize the different anatomical variants according to the Huang classification.

According to the Huang classification, our study showed type A1, 60.44%; type A2, 11.76%; type A3, 11.73%; type A4, 5.47%; type A5, 0.26%; and type B, which was identified in insignificant numbers (0.16%) or does not appear; additionally, variants that do not fit into the Huang classification have also been identified (10.18%).

The Huang classification serves as an invaluable presurgical guide, aiding in the strategic planning of biliary interventions and effectively reducing the risk of iatrogenic complications, morbidity, mortality, and postoperative length of stay. MRC is still considered the noninvasive gold standard method for evaluating the bile ducts and their anatomical variations.

## Introduction and background

Identifying the anatomical variations of the bile ducts preoperatively can help in the planning of surgical intervention and the prevention of complications associated with iatrogenic injuries. There are several classifications for these anatomical variants, some of which include Nakamura [1], Couinaud [2], Choi [3], Varotti [4], and Ohkubo [5], which classify the most variants, and Bageacu [6], Yoshida [7], Lee [8], Puente [9], Cho [10], Cucchetti [11], and Huang [12-18], which are the most commonly used in the literature related to imaging.

Every classification system aims to encompass the anatomical variants of the bile ducts, presenting their respective advantages and disadvantages. However, it is important to note that none of these systems can fully encompass all the variants described in the literature [2]. It is therefore necessary to standardize an identification method, at least for diagnosis, to provide detailed anatomical information that allows the surgeon to be guided in a timely manner prior to the surgical procedure.

## Review

Methods

**Figure 1:**
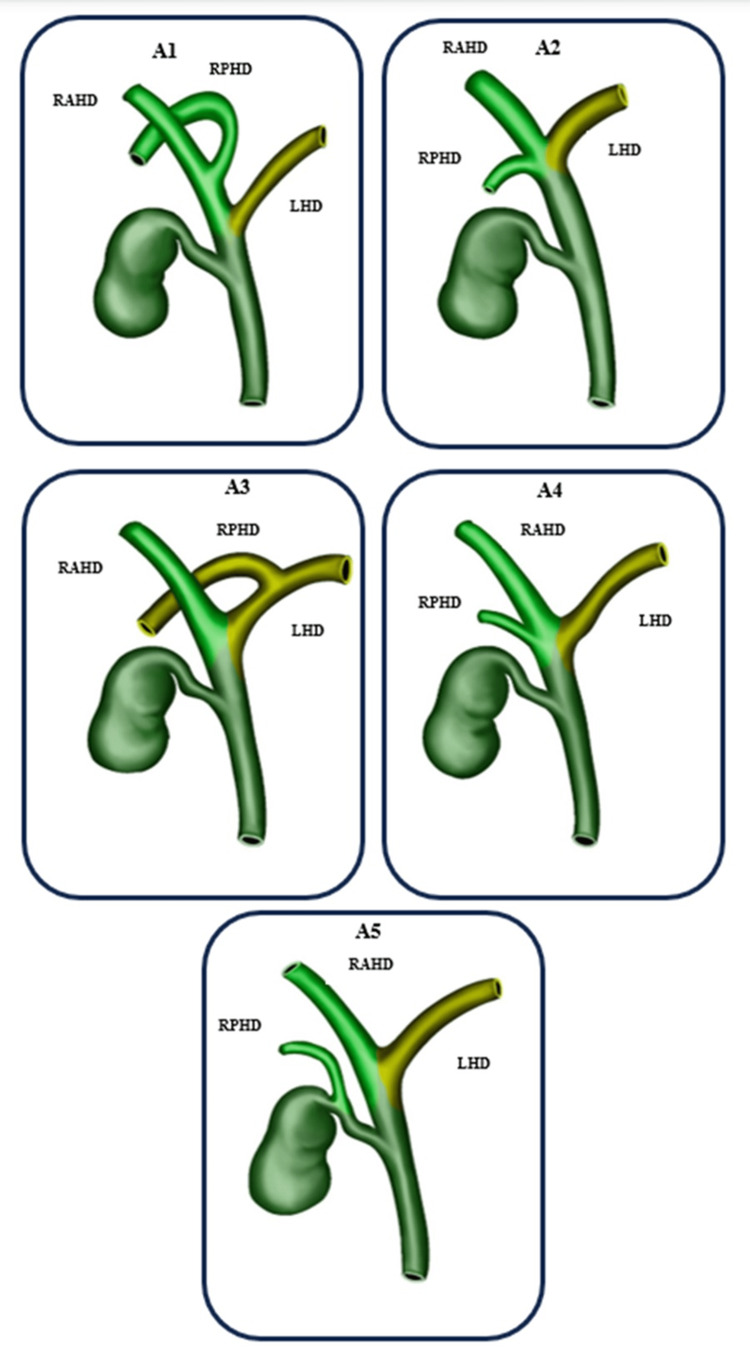
The Huang classification of the right hepatic duct Image produced by Rafael Lamparelli The Huang classification used for the right hepatic duct (RHD) anatomy is divided according to the insertion of the right posterior hepatic duct (RPHD) and the right anterior hepatic duct (RAHD). Type of classification: A1, A2, A3, A4, and A5 LHD: left hepatic duct

**Figure 2:**
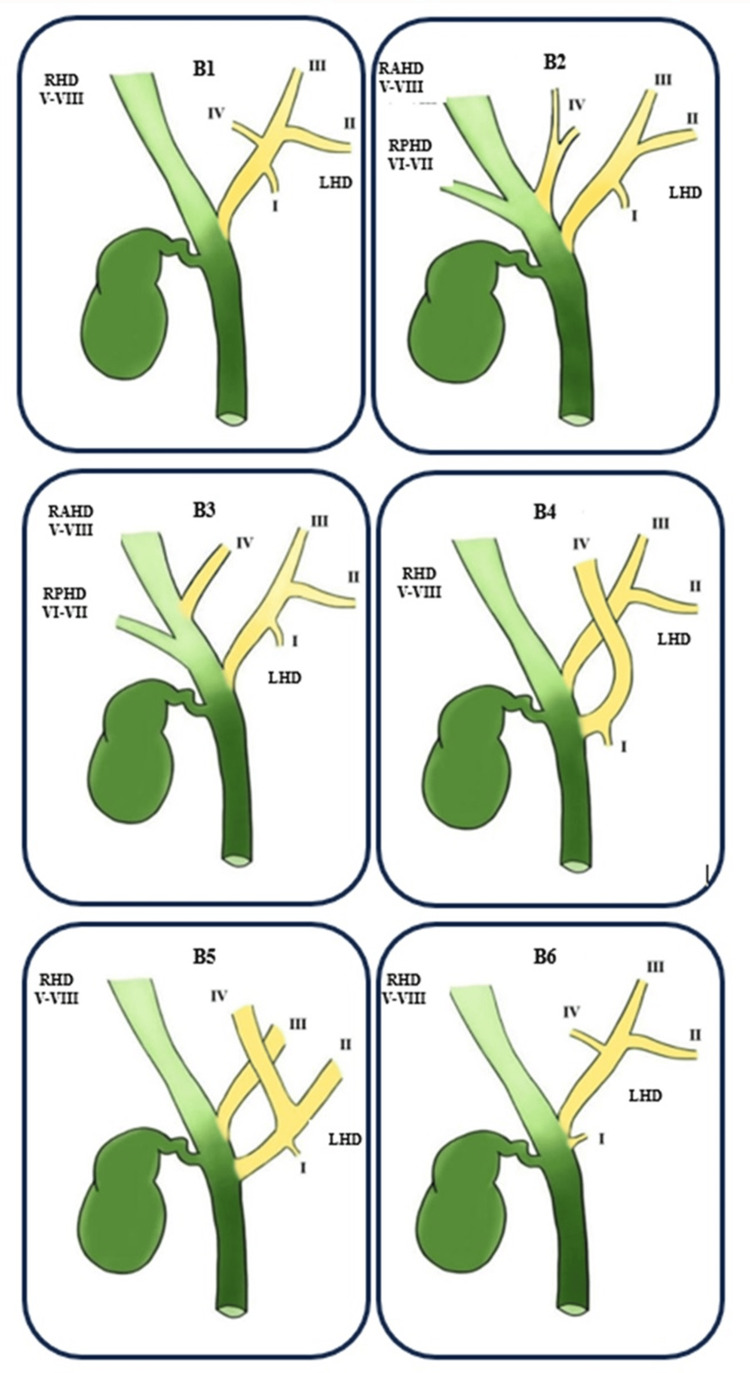
The Huang classification of the left hepatic duct (LHD) Image produced by Rafael Lamparelli The anatomy of the left hepatic duct (LHD), following the Huang classification, is divided according to the insertion of liver segments II-IV. Liver segments draining into the duct: I, II, III, IV, V, VI, VII, and VIII. Type of classification: B1, B2, B3, B4, B5, and B6 RHD, right hepatic duct; RPHD, right posterior hepatic duct; RAHD, right anterior hepatic duct

A review of the literature was carried out using the Cochrane Library database and the PubMed, Medical Literature Analysis and Retrieval System Online (MEDLINE), and Google Scholar platforms. The search terms used were "bile ducts," "bile ducts/injuries," "magnetic resonance imaging," and "anatomy." The pertinent studies were retrieved and subjected to detailed review. The Huang classification used for the right hepatic duct (RHD) anatomy is divided according to the insertion of the right posterior hepatic duct (RPHD) and the right anterior hepatic duct (RAHD) shown in Figure 1. The anatomy of the left hepatic duct (LHD), following the Huang classification, is divided according to the insertion of segments II-IV shown in Figure 2. Additionally, the identification methods used for the bile ducts and their relevance to the surgical procedure were also thoroughly examined.

Results

Table 1 displays the prevalence of anatomical variants in various populations based on the widely used Huang classification for the imaging evaluation of the bile duct anatomy [15].

**Table 1:** Distribution of anatomical variants by region The bold entries indicate the subtotal distribution by continent *Global total

Country	n	Huang classification													
		A1, n	%	A2, n	%	A3, n	%	A4, n	%	A5, n	%	B5, n	%	Other classifications, n	%
Spain	25	9	36.00	2	8.00	3	12.00	8	32.00	2	8.00	1	4.00	0	0.00
Italy	200	129	64.50	28	14.00	24	12.00	16	8.00	0	0.00	0	0.00	3	1.50
Germany	18	2	11.11	2	11.11	4	22.22	5	27.78	1	5.56	0	0.00	5	27.78
Greece	73	48	65.75	7	9.59	4	5.48	13	17.81	1	1.37	0	0.00	0	0.00
Europe	316	188	59.49	39	12.34	35	11.08	42	13.29	4	1.27	1	0.32	8	2.53
Egypt	120	79	65.83	14	11.67	16	13.33	9	7.50	2	1.67	0	0.00	0	0.00
Africa	120	79	65.83	14	11.67	16	13.33	9	7.50	2	1.67	0	0.00	0	0.00
Turkey	79	29	36.71	27	34.18	16	20.25	7	8.86	0	0.00	0	0.00	0	0.00
Palestine	342	309	90.35	29	8.48	2	0.58	1	0.29	1	0.29	0	0.00	0	0.00
Jordan	120	82	68.33	10	8.33	15	12.50	0	0.00	0	0.00	0	0.00	13	10.83
Saudi Arabia	177	104	58.76	19	10.73	12	6.78	32	18.08	2	1.13	0	0.00	8	4.52
Iran	362	163	45.03	78	21.55	48	13.26	13	3.59	0	0.00	0	0.00	60	16.57
India	253	134	52.96	29	11.46	46	18.18	18	7.11	0	0.00	6	2.37	20	7.91
Thailand	163	106	65.03	28	17.18	15	9.20	9	5.52	0	0.00	0	0.00	5	3.07
Taiwan	462	304	65.80	42	9.09	60	12.99	41	8.87	0	0.00	0	0.00	15	3.25
Japan	110	80	72.73	6	5.45	13	11.82	5	4.55	0	0.00	0	0.00	6	5.45
Japan	60	38	63.33	14	23.33	7	11.67	1	1.67	0	0.00	0	0.00	0	0.00
South Korea	300	188	62.67	29	9.67	34	11.33	19	6.33	6	2.00	4	1.33	20	6.67
South Korea	33	25	75.76	1	3.03	3	9.09	0	0.00	1	3.03	0	0.00	3	9.09
Asia	2461	1562	63.47	312	12.68	271	11.01	146	5.93	10	0.41	10	0.41	150	6.10
Canada	30	17	56.67	1	3.33	9	30.00	2	6.67	1	3.33	0	0.00	0	0.00
USA	108	78	72.22	6	5.56	0	0.00	7	6.48	1	0.93	0	0.00	19	17.59
Caribbean	152	109	71.71	29	19.08	7	4.61	6	3.95	0	0.00	0	0.00	1	0.66
Chile	3845	2217	57.66	426	11.08	487	12.67	177	4.60	0	0.00	0	0.00	538	13.99
Americas	4135	2421	58.55	462	11.17	503	12.16	192	4.64	2	0.05	0	0.00	558	13.49
Total*	7032	4250	60.44	827	11.76	825	11.73	389	5.53	18	0.26	11	0.16	716	10.18

Data was found available for the following regions: Spain [19], Italy [11], Germany [20], Greece [21], Egypt [22], Turkey [18], Palestine [23], Jordan [7], Saudi Arabia [24], Iran [14], India [25], Thailand [26], Taiwan [27], Japan [5,10], South Korea [3,28], Canada [29], the USA [8], Caribbean [17], and Chile [9]. The different classifications presented by the authors were translated into the Huang classification and listed in Tables 2-8.

**Table 2:** Distribution of anatomical variants of the Huang classification type A1 "Classification of other authors" corresponds to the following classifications used by other authors: Couinaud [19,21], Cucchetti [11], Ohkubo [5,20], Huang [14,17,18,22,29], Abdelkareem [23], Yoshida [7,27], Al-Jiffry [24], Sharma [25], Thungsuppawattanakit [26], Cho [10], Choi [3,28], Lee [8], and Puente [9]. "Type" corresponds to the variant type in each classification

Classification of other authors	Type	n	Rate by classification of other authors
Couinaud [19,21]	A	57	58.16
Cucchetti [11]	1	129	64.50
Ohkubo [5,20]	A and D	82	64.06
Huang [14,17,18,22,29]	A1	397	53.43
Abdelkareem [23]	a and b	309	90.35
Yoshida [7,27]	1	386	66.32
Al-Jiffry [24]	A	104	58.76
Sharma [25]	1	134	52.96
Thungsuppawattanakit [26]	A	106	65.03
Cho [10]	1	38	63.33
Choi [3,28]	1	213	63.96
Lee [8]	Typical anatomy	78	72.22
Puente [9]	1	2217	57.66

**Table 3:** Distribution of anatomical variants of the Huang classification type A2 "Classification of other authors"corresponds to the following classifications used by other authors: Couinaud [19,21], Cucchetti [11], Ohkubo [5,20], Huang [14,17,18,22,29], Abdelkareem [23], Yoshida [7,27], Al-Jiffry [24], Sharma [25], Thungsuppawattanakit [26], Cho [10], Choi [3,28], Lee [8], and Puente [9]. "Type" corresponds to the variant type in each classification

Classification of other authors	Type	n	Rate by classification of other authors
Couinaud [19,21]	B	9	9.18
Cucchetti [11]	2	28	14.00
Ohkubo [5,20]	B	8	6.25
Huang [14,17,18,22,29]	A2	149	20.05
Abdelkareem [23]	c	29	8.48
Yoshida [7,27]	2	52	8.93
Al-Jiffry [24]	B	19	10.73
Sharma [25]	2	29	11.46
Thungsuppawattanakit [26]	B	28	17.18
Cho [10]	2	14	23.33
Choi [3,28]	2	30	9.01
Lee [8]	Trifurcation	6	5.56
Puente [9]	2	426	11.08

**Table 4:** Distribution of anatomical variants of the Huang classification type A3 "Classification of other authors" corresponds to the following classifications used by other authors: Couinaud [19,21], Cucchetti [11], Ohkubo [5,20], Huang [14,17,18,22,29], Abdelkareem [23], Yoshida [7,27], Al-Jiffry [24], Sharma [25], Thungsuppawattanakit [26], Cho [10], Choi [3,28], Lee [8], and Puente [9]. "Type" corresponds to the variant type in each classification

Classification of other authors	Type	n	Rate by classification of other authors
Couinaud [19,21]	D	7	7.14
Cucchetti [11]	3A	24	12.00
Ohkubo [5,20]	C	17	13.28
Huang [14,17,18,22,29]	A3	96	12.92
Abdelkareem [23]	d	2	0.58
Yoshida [7,27]	3	75	12.89
Al-Jiffry [24]	D1 and D2	12	6.78
Sharma [25]	3A	46	18.18
Thungsuppawattanakit [26]	D	15	9.20
Cho [10]	3	7	11.67
Choi [3,28]	3A	37	11.11
Lee [8]	Abnormal right configuration 1	0	0.00
Puente [9]	3	487	12.67

**Table 5:** Distribution of anatomical variants of the Huang classification type A4 "Classification of other authors" corresponds to the following classifications used by other authors: Couinaud [19,21], Cucchetti [11], Ohkubo [5,20], Huang [14,17,18,22,29], Abdelkareem [23], Yoshida [7,27], Al-Jiffry [24], Sharma [25], Thungsuppawattanakit [26], Cho [10], Choi [3,28], Lee [8], and Puente [9]. "Type" corresponds to the variant type in each classification

Classification of other authors	Type	n	Rate by classification of other authors
Couinaud [19,21]	C	21	21.43
Cucchetti [11]	3B	16	8.00
Ohkubo [5,20]	E	10	7.81
Huang [14,17,18,22,29]	A4	37	4.98
Abdelkareem [23]	e	1	0.29
Yoshida [7,27]	IV	41	7.04
Al-Jiffry [24]	C1 and C2	32	18.08
Sharma [25]	3B	18	7.11
Thungsuppawattanakit [26]	C	9	5.52
Cho [10]	4	1	1.67
Choi [3,28]	3B	19	5.71
Lee [8]	Abnormal right configuration 2	7	6.48
Puente [9]	4	177	4.60

**Table 6:** Distribution of anatomical variants of the Huang classification type A5 "Classification of other authors" corresponds to the following classifications used by other authors: Couinaud [19,21], Cucchetti [11], Ohkubo [5,20], Huang [14,17,18,22,29], Abdelkareem [23], Yoshida [7,27], Al-Jiffry [24], Sharma [25], Thungsuppawattanakit [26], Cho [10], Choi [3,28], Lee [8], and Puente [9]. "Type" corresponds to the variant type in each classification

Classification of other authors	Type	n	Rate by classification of other authors
Couinaud [19,21]	F	3	3.06
Cucchetti [11]		0	0.00
Ohkubo [5,20]		0	0.00
Huang [14,17,18,22,29]	A5	3	0.40
Abdelkareem [23]	F	1	0.29
Yoshida [7,27]		0	0.00
Al-Jiffry [24]	F	2	1.13
Sharma [25]	3C	0	0.00
Thungsuppawattanakit [26]		0	0.00
Cho [10]		0	0.00
Choi [3,28]	3C	7	2.10
Lee [8]	Abnormal right configuration 3	1	0.93
Puente [9]		0	0.00

**Table 7:** Distribution of anatomical variants of the Huang classification type B5 "Classification of other authors" corresponds to the following classifications used by other authors: Couinaud [19,21], Cucchetti [11], Ohkubo [5,20], Huang [14,17,18,22,29], Abdelkareem [23], Yoshida [7,27], Al-Jiffry [24], Sharma [25], Thungsuppawattanakit [26], Cho [10], Choi [3,28], Lee [8], and Puente [9]. "Type" corresponds to the variant type in each classification

Classification of other authors	Type	n	Rate by classification of other authors
Couinaud [19,21]	E1	1	1.02
Cucchetti [11]		0	0.00
Ohkubo [5,20]	J	0	0.00
Huang [14,17,18,22,29]	B5	0	0.00
Abdelkareem [23]		0	0.00
Yoshida [7,27]		0	0.00
Al-Jiffry [24]		0	0.00
Sharma [25]	6	6	2.37
Thungsuppawattanakit [26]		0	0.00
Cho [10]		0	0.00
Choi [3,28]	6	4	1.20
Lee [8]	Others	0	0.00
Puente [9]		0	0.00

**Table 8:** Distribution of anatomical variants not of the Huang classification "Classification of other authors" corresponds to the following classifications used by other authors: Couinaud [19,21], Cucchetti [11], Ohkubo [5,20], Huang [14,17,18,22,29], Abdelkareem [23], Yoshida [7,27], Al-Jiffry [24], Sharma [25], Thungsuppawattanakit [26], Cho [10], Choi [3,28], Lee [8], and Puente [9]. "Type" corresponds to the variant type in each classification

Classification of other authors	Type	n	Rate by classification of other authors
Couinaud [19,21]	D2 and E2	0	0.00
Cucchetti [11]	Others	3	1.50
Ohkubo [5,20]	F, G, H, I, K, and Others	11	8.59
Huang [14,17,18,22,29]		61	8.21
Abdelkareem [23]		0	0.00
Yoshida [7,27]	Others	28	4.81
Al-Jiffry [24]	E	8	4.52
Sharma [25]	4, 5, and 7	20	7.91
Thungsuppawattanakit [26]	Others	5	3.07
Cho [10]		0	0.00
Choi [3,28]	Others	23	6.91
Lee [8]	Others	16	14.81
Puente [9]	Others	538	13.99

In our study, the distribution of the variants was presented as follows: type A1, the RPHD and RAHD unite to form the RHD (60.44%); type A2, the RHD is absent, and the RPHD, RAHD, and the left hepatic duct join to form the common hepatic duct (CHD) (11.76%); type A3, the RPHD or RAHD connects directly to the LHD (11.73%); type A4, the RPHD or RAHD connects directly to the CHD (5.47%); type A5, the RPHD communicates to the cystic duct or its periphery in an aberrant way and others (such as the union of the accessory duct to the CHD and to the RHD) (0.26%); type B1, segment IV connects to the LHD (not present in our review); type B2, segment IV connects to the CHD, separate from segments II and III (not present in our review); type B3, segment IV connects to the RAHD (not present in our review); type B4, segment IV connects to the CHD (not present in our review); and type B5, segment IV connects to segment II or other segments (such as connecting segments II and III directly to the RHD or CHD {0.16%}). More complex variants that include accessory ducts do not follow the Huang classification, and in the reviewed literature, they have a prevalence of 10.18%.

**Figure 3:**
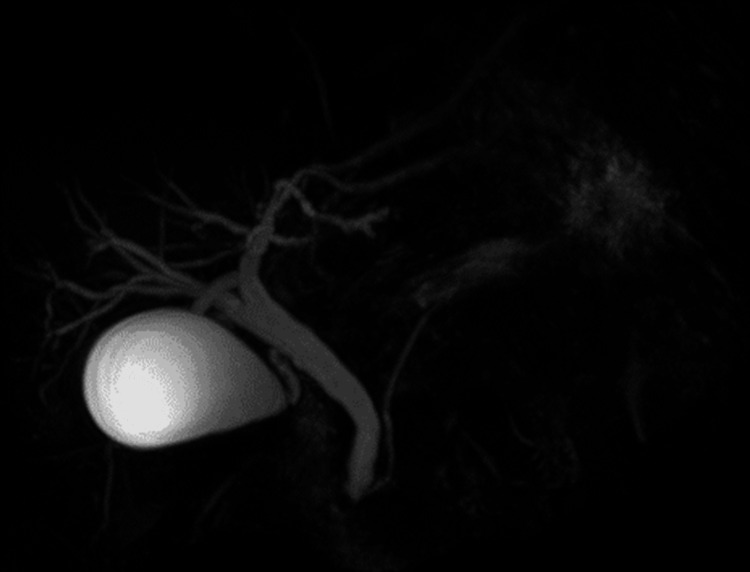
MRC Image produced by Norman Olmedo MRC: magnetic resonance cholangiography

While the identification of biliary anatomy can be achieved by different diagnostic methods, currently, magnetic resonance cholangiography (MRC) has proven to be the most useful tool for this purpose (Figure 3) due to its safety, as it is a noninvasive technique with a high degree of sensitivity and specificity ranging from 93% to 100% [20,22].

Although the surgical complication rate of patients undergoing biliary surgery is relatively low (0.12%-0.59%) [14], having a useful tool in the identification of anatomical variants greatly facilitates surgical planning.

Discussion

The literature reviewed shows that, in the entire population (n = 7032), there is a higher incidence of type A1 morphology according to the Huang classification, encompassing 60.44% of all cases, followed by variant type A2 (trifurcation between RPHD, RAHD, and LHD), with 11.76%, and A3, with 11.73%.

LHD variants are less common. In our review, we identified 0.16% of the variants of type B5 according to the Huang classification. However, other authors with large study populations have managed to sample other types of variants of the left hepatic duct; for example, Chaib et al. [13] reported the frequency of LHD variations in 1014 patients according to the Huang classification, as follows: type B1, 773 (76.2%); type B2, 153 (15%); type B3, 38 (3.7%); types B4, nine (0.8%); and type B5, 29 (2.8%).

The distributions of variants in the bile duct by region in our study (Europe, Africa, Asia, and America) were similar to those in A1, A2, and A3 in the proposed Huang reclassification; however, there was some variation between regions in the distribution of variant type B in the left hepatic duct, which was consistent with the findings from small samples of the populations studied.

These variants of both RHD and LHD represent a certain risk for patients with biliary pathology that can be resolved surgically. In laparoscopic and open cholecystectomies, complications related to bile duct injuries may occur. The frequency with which it occurs is 0.59% and 0.125%, respectively [30]. The high number of hepatobiliary surgeries and the greater current tendency to perform laparoscopic surgery led to the adoption of guidelines where some aspects were considered strategically to minimize the risk of bile duct injuries and potentially severe consequences that derive from these [31].

Among the risk factors for iatrogenic lesions of the bile ducts are anatomical variants, in addition to those inherent to the laparoscopic technique, which includes inadequate training [32]. Therefore, classifying the anatomical variant of the bile ducts prior to surgery is important. Fluorescent cholangiography using indocyanine green (ICG-C) prior to surgery allows the visualization of the structures of the biliary tree and thus minimizes the risk of lesions in the bile ducts. Fluorescent cholangiography using ICG is performed because intravenously injected ICG passes entirely through the bile ducts and emits light at a wavelength of 830 nm in the near-infrared region. One milliliter of ICG (2.5 mg/mL) is administered intravenously 30 minutes before the patients enter the operating room. The maximum action time is two hours, at which point the fluorescence allows the identification of the bile ducts to avoid injury [33].

Intraoperative cholangiography (IC) is another technique used to visualize the anatomy of the bile ducts while performing a laparoscopic procedure. IC reduces the morbidity associated with biliary tract injury, provides a clear depiction of the biliary anatomy, and provides critical insight into safety by helping surgeons identify the cystic duct before division. Additionally, it offers intraoperative information for identifying stones in the common bile duct, enabling their removal during surgery and reducing subsequent episodes of cholangitis and pancreatitis [34].

The disadvantages of intraoperative cholangiography include the following: requires excessive time to perform, patients and medical staff are exposed to radiation, requires additional human and material resources, and can cause injury to the bile ducts because it requires the insertion of a cannula for contrast agent injection [35].

Magnetic resonance (MR) cholangiography (MRC) is a special noninvasive type of magnetic resonance imaging (MRI) that uses a strong magnetic field and radio waves to produce a detailed image of the biliary tree with the capacity to show the presence of biliary stones with a sensitivity of 85%, specificity of 93%, positive predictive value (PPV) of 87%, and negative predictive value (NPV) of 82%, according to Griffin et al. [36]. For the evaluation of biliary anatomy, studies have also reported high sensitivity, specificity, positive predictive value (PPV), and negative predictive value (NPV) above 93%, reaching up to 100%, especially for specificity [29,37].

A variant of this technique is the functional MRC, where an intravenous paramagnetic contrast agent is used. On magnetic resonance imaging (MRI), using a specific hepatobiliary contrast agent, healthy hepatocytes take up and process the contrast agent, which is eliminated in the bile. The paramagnetic properties of the contrast agent cause a decrease in the longitudinal relaxation time (T1) of the liver and biliary tree. Examples of this type of contrast are as follows: mangafodipir trisodium (Teslascan), gadobenate dimeglumine (MultiHance), and gadoxetic acid-gadolinium-ethoxybenzyl-diethylenetriamine penta-acetic acid (Primovist or Eovist).

These contrast agents can be administered at different doses and with different pharmacodynamics [38,39]. Functional MRC is more expensive than the conventional technique, the diagnostic certainty is similar, and it works only for the biliary tree; therefore, it is not very widespread. The advantages of the functional MRC include the following: better demonstration of intercommunications and congenital anomalies in the bile ducts, delayed biliary excretion to be distinguished from nonexcretion, demonstration of active bile leakage, dynamic analysis of pre-contrast and post-contrast images [36].

MRC without a hepatospecific contrast agent or conventional MRC is currently considered the gold standard for the evaluation of hepatobiliary disease and anatomical variations of the bile ducts. Bile duct exploration techniques generally include T2-weighted sequences with extremely long echo times, thus essentially achieving a hyperintense representation of liquid content. The two most used techniques are T2-weighted two-dimensional (2D) single-shot and three-dimensional (3D) turbo spin echo methods, the latter of which have greater precision for revealing anatomical details of the biliary tree [38]. In addition to being noninvasive, this method does not use contrast agents and is free of complications [40-42]. The combination of two of the three magnetic resonance imaging (MRI) methods (conventional MRC, three-dimensional MRI, and functional MRC) allows for a significant increase in diagnostic certainty and predictive confidence compared with the use of only one [37].

According to Strasberg, bile duct injury is a serious and common complication of cholecystectomy; therefore, to address this surgical procedure, he proposes ensuring the safe identification of key anatomical structures, making the appropriate decision to avoid performing a total cholecystectomy in situations where it is too risky to achieve secure identification, and safely completing surgery when anatomical identification of key structures is not possible [43].

There are risk factors for complications associated with both laparoscopy and laparotomy surgery. Complications that occur during cholecystectomy can be evaluated according to a severity scale ranging from 1 to 4: deviation from the ideal postoperative course (grade 1), life-threatening (grade 2a), life-threatening/residual disability/requirement for invasive procedures such as surgery (grade 2b), life-threatening/residual disability/requirement for invasive procedures (surgery)/organ resection or life-threatening persistence (grade 3), and death due to complications (grade 4) [44].

According to Clavien et al. [44], the advantages of this classification are as follows: greater uniformity in the reporting of results, possibility of being used to compare results in the same center in different periods of time, ability to compare different centers, ability to compare the results of surgical to nonsurgical measures, usability for proper meta-analyses, objective identification of preoperative risk factors, and establishment of preoperative forecasts.

Severity rating scales have also been used to compare the complications of laparoscopic and open cholecystectomy, and laparoscopic cholecystectomy is considered ideal for elective surgeries due to its lower possibility of complications [45,46]. Since the 1990s, the Clavien classification has been used as a severity rating scale to identify complications in laparoscopic cholecystectomy, although with lower use in recent years [47].

With the preferential adoption of laparoscopic cholecystectomy, the incidence of surgical complications such as bile duct injuries has increased. This incidence has decreased over time, and it is now 2-3 times more common to find bile leakage as a complication of laparoscopic cholecystectomy. Mortality in the first year after surgery is three times higher for bile duct lesions than for bile leakage [48]. The main cause of bile duct injuries as a complication of laparoscopic cholecystectomy is the misidentification of anatomical variants of the bile ducts [49].

Although no reports of any specific type of anatomical variant related to a certain surgical complication have been found in the reviewed literature, this seems to be related to the lack of relevant cases; however, certain variants, such as short RHD, can predict the need for more complex surgical procedures, such as bench ductoplasty or multiple anastomoses. In this sense, the studies conducted thus far are insufficient to investigate whether variations in the biliary tree affect the outcomes and the development of procedures such as laparoscopic or open cholecystectomy and whether this increases the risk of bile duct injury [50].

However, an abnormal bile duct pathway is still considered the most important anatomical factor. That is, compared to extrahepatic bile ducts that have normal routes, intraoperative injury to the hepatic duct occurs 3.2-8.4 times more frequently in patients with extrahepatic bile ducts with some type of anatomical variant, whether open cholecystectomy or laparoscopic cholecystectomy is performed [50].

## Conclusions

The Huang classification is one of the most commonly used systems for characterizing anatomical variants of the bile ducts. Taking this classification into account, type A1 is the most prevalent. Anatomical variations determine the risk of surgical complications due to iatrogenic injuries to the bile ducts. MRC with specific hepatobiliary contrast is useful for visualizing anatomical variants of the bile ducts, although this technique is surpassed by fluorescent cholangiography with indocyanine green (ICG). Nonetheless, noncontrast or conventional MRI are currently considered the gold standard for detecting hepatobiliary disease and anatomical variations in the bile ducts, with the three-dimensional MRC technique being the most accurate for revealing the details of the biliary tree. The most frequent complications of laparoscopic cholecystectomy are bile duct injuries and bile leakage, the former being the complication that has the highest mortality.
